# Should Soft Tissue Sarcomas be Treated at a Specialist Centre?

**DOI:** 10.1080/13577140410001679185

**Published:** 2004-03

**Authors:** A. A. Bhangu, J. A. S. Beard, R. J. Grimer

**Affiliations:** 1 University of Birmingham Medical School Edgbaston Birmingham B15 2TT UK; 2 Royal Orthopaedic Hospital NHS Trust Woodlands Northfield Birmingham B31 2AP UK

## Abstract

*Objective*. We have investigated whether there is evidence that patients with soft tissue sarcomas do better if treated in a specialist centre compared with district general hospitals.

*Patients*. All patients diagnosed with soft tissue sarcomas who were residents of WMRHA between 1994 and 1996, with minimum follow up of 5 years, excluding head and neck or retroperitoneal tumors.

*Methods*. We reviewed data from the Royal Orthopaedic Hospital Oncology Service (ROHOS) database and the Cancer Intelligence Unit (CIU) Database, with medial record review where necessary. Main outcome measures were local recurrence and overall survival.

*Results*. A total of 260 patients were diagnosed as having STS over the 3-year period (incidence=1.62per 100000 per year): 37% of patients had the majority of treatment at the specialist centre under the care of three surgeons, whilst the other 63% were treated at a total of 38 different hospitals. The rate of local recurrence was 39% at the district general hospitals compared with 19% at the specialist centre despite the fact that tumours treated at the district hospitals were smaller and of lower grade. The most significant factors affecting survival were grade (high versus low) and depth of the tumour. Patients treated at the specialist centre had a small survival advantage after multivariate testing.

*Conclusion*. Soft tissue sarcomas are rare. Centralization of treatment improves local control in all patients and survival in some. Appropriate mechanisms for ensuring that patients with soft tissue sarcomas are seen and treated at specialist centres should be developed.


*Sarcoma*, March 2004, *VOL. 8, NO. 1, 1–6*

Taylor&Francis healthsciences

ORIGINAL ARTICLE

### Should soft tissue sarcomas be treated at a specialist centre?

A.A. BHANGU[1], J.A.S BEARD[1] & R.J. GRIMER[2]

[1]*University of Birmingham Medical School, Edgbaston, Birmingham, B15 2TT, UK &* [2]*Royal Orthopaedic Hospital NHS Trust, Woodlands, Northfield, Birmingham, B31 2AP, UK*

**Abstract**
*Objective*. We have investigated whether there is evidence that patients with soft tissue sarcomas do better if treated in a specialist centre compared with district general hospitals.
*Patients*. All patients diagnosed with soft tissue sarcomas who were residents of WMRHA between 1994 and 1996, with minimum follow up of 5 years, excluding head and neck or retroperitoneal tumors.
*Methods*. We reviewed data from the Royal Orthopaedic Hospital Oncology Service (ROHOS) database and the Cancer Intelligence Unit (CIU) Database, with medial record review where necessary. Main outcome measures were local recurrence and overall survival.
*Results*. A total of 260 patients were diagnosed as having STS over the 3-year period (incidence=1.62per 100000 per year):37% of patients had the majority of treatment at the specialist centre under the care of three surgeons, whilst the other 63% were treated at a total of 38 different hospitals. The rate of local recurrence was 39% at the district general hospitals compared with 19% at the specialist centre despite the fact that tumours treated at the district hospitals were smaller and of lower grade. The most significant factors affecting survival were grade (high versus low) and depth of the tumour. Patients treated at the specialist centre had a small survival advantage after multivariate testing.
*Conclusion*. Soft tissue sarcomas are rare. Centralization of treatment improves local control in all patients and survival in some. Appropriate mechanisms for ensuring that patients with soft tissue sarcomas are seen and treated at specialist centres should be developed.

**Key words:** *tumour, soft tissue sarcoma, specialist centre*

### Introduction

'Cancer should be treated by cancer specialists' is often stated, but there is little proof that outcomes are different. Soft Tissue Sarcomas (STS) represent less than one percent of all malignancies and due to such rarity, appropriate diagnosis, staging and treatment is difficult and widely varied outside of a specialist centre, due to lack of experience and familiarity.[1,2] This is largely due to the lack of realization that a lump may be malignant despite published guidelines highlighting the features of potential malignancy.[3]

It is recommended that any lump that is either bigger than 5cm, deep to the fascia, increasing in size or painful should be considered to potentially be a sarcoma and the patient should be referred to an appropriate specialist centre before biopsy or surgery.[4–6] This enables co-ordinated, specialist care by a multidisciplinary team comprising of orthopaedic oncology surgeons, pathologists and radiotherapists, achieving wide margins at first attempt, thus reducing risk of local recurrence and further surgery.[7,8]

Proving that centralization of care produces better results is not, however, easy although there is some information available for more common cancers, such as breast, colorectal and oral cancer.[9–11] We have investigated whether there is any difference in outcome for patients with STS treated at a specialist centre or a non-specialist District General Hospital (DGH).

### Patients and methods

We analysed the outcomes for all patients diagnosed with soft tissue sarcomas in one health region of the UK over a 3-year period, with minimum follow up of 5 years. The region has a population base of 5306497 (2620859 men and 2685638 women–1995 figures) and has 38 District General Hospitals. Only one

Correspondence to: R.J. Grimer, Orthopaedic Hospital NHS Trust, Woodlands, Northfield, Birmingham, B31 2AP, UK. Tel.: +44-121-685-4150; Fax: +44-121-685-4146; E-mail: rob.grimer@roh.nhs.uk

1357-714X print/1369–1643 © 2004 Taylor & Francis Ltd
DOI: 10.1080/13577140410001679185

hospital had a unit with a multidisciplinary team managing sarcomas during this time period and this was designated the 'specialist centre' (SC).

Potentially eligible patients were initially identified from the Cancer Intelligence Unit database. We included any patient with a newly diagnosed STS during the period of 1/1/1994 to 31/12/1996. Patients with head and neck, GIST or retroperitoneal sarcomas were excluded. The CIU database contained information on diagnosis, treatment and outcomes for all patients with cancer in the Region and is particularly reliable for death certification. Patients who had their definitive treatment at the SC were identified from the SC prospective patient database and information from the two sources was used in subsequent evaluations. In cases where there was incomplete information reference was made to the original patient records from the appropriate hospital. Patients who were referred to the SC at a later stage of their treatment (e.g., when they developed local recurrence) were still included in the DGH group as that was where they had their initial treatment, but patients who were referred to the SC after biopsy or excision biopsy were judged to have had their definitive treatment at the Specialist Centre.

Ethical Committee Approval was obtained from the Regional Multi-centre Research Ethical Committee (MREC) for use of both databases, and a letter sent to the Caldicott Guardian of each NHS Trust explaining that data would be used to analyse specialist versus non-specialist treatment only, not to criticize individual surgeons or Trust performance.

### *Statistical methods*

Demographic details including the distribution of age, size, grade and depth of STS are described along with treatment variables. Difference between groups were assessed using the Chi-squared test or *t*-test. Overall survival was calculated using Kaplan–Meier survival curves and the impact of prognostic factors was assessed using the log-rank.[12,13] Multivariate analysis was performed using Cox's proportional hazard method with variables being chosen using a forward conditional stepwise approach. Relative risks have been calculated using a proportional hazards model with only the noted covariate in the model. Significance was set at $P<0.05$ for two-sided tests. Survival time was calculated from the time of diagnosis when investigating the significance of tumour and patient characteristics. The end point was taken as time of death or the last documented time the patient was known to be alive. Patients who died of unrelated causes were censored at the time of their death. Analyses were performed using Statview.[14] When factor analysis was undertaken the numbers involved have been highlighted.

### *Patients*

A total of 116 patients were identified on the SC database as being treated for soft tissue sarcoma (excluding retroperitoneal and Kaposi's Sarcomas) between 1994 and 1996 inclusive and living in the Region. Of these, 15 were identified to have received treatment before 1994 and were thus excluded from the study. Four were identified as having been referred from a DGH for local recurrence within the time period, and were found to be already included on the DGH list. Patients referred for re-excision, following inadvertent or incomplete excision, were included on the SC list. One patient was lost to follow-up. This resulted in the SC list being 96 patients (117−(15+4+1+1)=96).

A total of 213 patients were identified by the CIU as having been treated at a non-specialist centre and residing in the region (excluding Kaposi's sarcomas). Of these, 42 were sarcomas of retroperitoneal origin and were thus excluded. Five patients were identified as being incorrectly classified as having STS, and two had been treated before 1994. This resulted in DGH list being 164 (213−(42+5+2)=164).

The overall total sample size was thus 260 patients over a 3-year period, resulting in an age-standardised incidence ratio of 1.62per 100000 per year.

### Results

### *Demographics*

The distribution of patient and tumour characteristics, split by centre of treatment is shown in Table 1. The median age of the patients was 61 years with a broad range (Fig. 1). 29 patients (11%) had metastases at diagnosis, with the proportion at both DGH and SC being the same. The prognosis for these patients was awful (median survival 4 months) and as a result these patients had been excluded from all subsequent analyses of both treatment and outcome.

Table 1 shows that patients treated at the specialist centre tended to be younger and they had larger tumours with a greater proportion of both deep and high grade tumours. The most common diagnosis was leiomyosarcoma (27%) followed by liposarcoma (20%) and the most common site was in the thigh. There was a significantly greater proportion of patients with UICC (Union Internationale Contre le Cancer) stage 3 tumours (high grade, deep, >5cm) treated at the specialist centre than at the DGHs ($P=0.0003$).[15]

### *Treatment*

All patients without metastases at diagnosis underwent excision of their STS with curative intention. Amputation was the primary procedure in 8% of

**Table 1.** *Patient and treatment factors, split by centre*

| Factor | Total | DGH ($n$=164) | SC ($n$=96) | $P$ value |
|---|---|---|---|---|
| Mean (median) age | 57 (61) | 58 (62) | 54 (61) | NS |
| Sex ratio (M/F) | 149/111 | 91/73 | 58/38 | NS |
| Mean size (cm)(median) | 8.5 (7.0) | 7.5 (4.8) | 10.3 (9.0) | 0.003 |
| Proportion>5cm | 57% | 46% | 73% | 0.0001 |
| Proportion of deep tumors | 65% | 60% | 72% | NS |
| Proportion of high grade tumours | 71% | 69% | 79% | 0.05 |
| Metastases at presentation | 11% | 12% | 11% | NS |
| Large, high grade, deep tumours (UICC Stage 3) | 31% | 21% | 45% | 0.0003 |

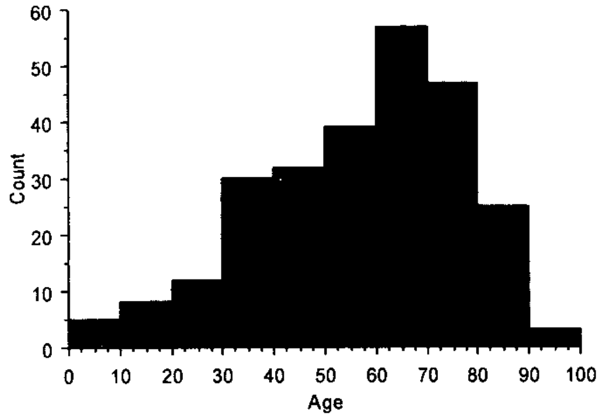

**Fig. 1.** *Age distribution of the 260 patients.*

cases with the other 92% having limb salvage surgery. In cases where the margins of excision were documented, adequate excision margins (a wide or radical margin) were achieved in 37% of cases (35% of DGH cases and 39% of SC cases).

*Outcomes*

*Local control*

Local recurrence arose in 73 patients (31%). It was related to centre of treatment but not to the size of the tumour, depth or grade. (Fig. 2, Table 2). Patients with an adequate excision had a LR rate of 26% compared to a 40% risk in patients with an inadequate excision. This was not however a significant difference ($P$=0.10). When local recurrence was stratified by centre of treatment there was a highly significant difference between the centres for all the above parameters apart from small and low grade tumours (Table 2).

*Overall survival*

The overall survival rate of the non metastatic patients was 58% at 5 years and did not appear to be significantly different between the two centres (Fig. 3). Twenty patients died of unrelated causes since the time of diagnosis. Apart from being predominantly older than 60 there was no other particular predominance of clinical features in this

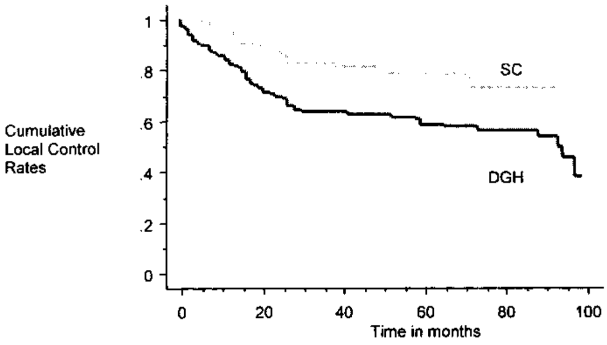

**Fig. 2.** *Kaplan–Meier graph showing local control rates split by centre of treatment (P=0.0064).*

**Table 2.** *Outcome measures: local recurrence rates*

| Factor | Total (%) | DGH (%) | SC (%) | $P$ value |
|---|---|---|---|---|
| All patients | 32 | 39 | 19 | 0.0011 |
| Subcutaneous | 31 | 40 | 15 | 0.023 |
| Deep | 32 | 41 | 21 | 0.016 |
| Large (>5cm) | 30 | 45 | 21 | 0.0009 |
| Small (<5cm) | 33 | 37 | 16 | 0.15 |
| High grade | 33 | 41 | 20 | 0.0045 |
| Low grade | 29 | 35 | 12 | 0.06 |
| Adequate margins | 26 | 39 | 12 | 0.025 |
| Inadequate margins | 40 | 45 | 33 | 0.21 |
| UICC stage 3 | 28 | 46 | 17 | 0.01 |
| Others | 31 | 38 | 18 | 0.018 |

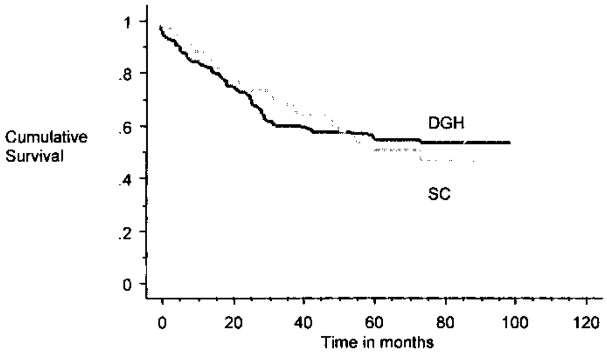

**Fig. 3.** *Kaplan–Meier curve of overall survival comparing the two different treatment centres.*

**Table 3.** *Overall survival: univariate and multivariate analysis*

| Factor | Univariate | | | Multivariate | | |
|---|---|---|---|---|---|---|
| | Hazard ratio | Confidence limits | *P* value | Hazard ratio | Confidence limits | *P* value |
| DGH | 0.96 | 0.63–1.47 | 0.84 | 1.7 | 1.01–2.8 | 0.048 |
| Age>60 | 1.90 | 1.24–2.90 | 0.0029 | | | NS |
| High grade | 3.60 | 1.92–6.81 | <0.0001 | 6.24 | 2.64–14.63 | <0.0001 |
| Deep | 3.94 | 2.17–7.16 | <0.0001 | 3.10 | 1.64–5.84 | 0.0005 |
| Small (<5cm) | 0.42 | 0.26–0.70 | 0.0008 | 0.52 | 0.30–0.92 | 0.024 |

group. Factors related to survival are shown in Table 3 using both univariate and multivariate analysis. This confirms the expected prognostic factors for disease related survival on univariate testing; i.e., size at presentation, depth, age and grade.

Because of the significantly higher proportion of patients with large, deep and high grade tumours treated at the SC we have included centre of treatment in the multivariate analysis, even though it was not found to be significant on univariate testing. This shows that whilst grade of tumour remains the most significant factor affecting survival, along with size and depth, the centre of treatment just remains significant in the multivariate model. Age loses its significance probably because of the increasing number of non-tumour-related deaths in this group. When analyzed by UICC state, however, the only significant difference between the two centres is for stage 3 tumours, all other stages showing no significant difference in overall survival (Fig. 3).

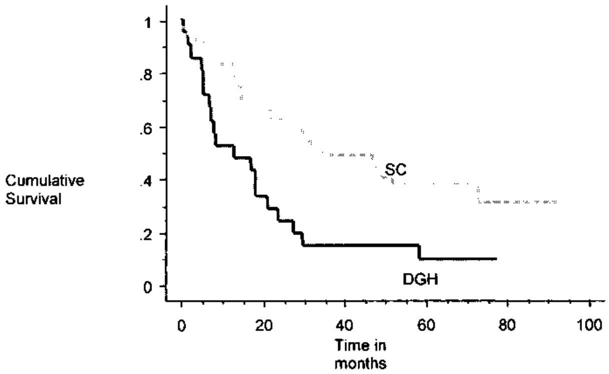

**Fig. 4.** *Kaplan–Meier survival curve for patients with large (>5cm), deep and high grade tumors only. The difference is significant (P=0.0032).*

### Discussion

The aim of our study was to carry out a survival analysis on patients treated at a specialist centre compared to non-specialist centres (38 District General Hospitals) in one Health Region in the UK.

We found similar proportions of patients with large, high grade and deep tumours to those identified in other population based studies.[4] We did, however, find that the tumours of patients treated at the specialist centre tended to be larger, with a greater proportion being both high grade and deep to the fascia. This is of importance as these three factors are well established prognostic factors for overall survival in STS.[16] The only factor carrying a worse prognosis which was over represented in the DGH population was older age (Fig. 4).

It is not surprising that patients with large and deep tumours were seen at the specialist centre — these are the lumps which should provoke most suspicion of malignancy and this does indicate that even in the time period under review (1994–1996) there was a tendency for these 'worrying' lumps to be referred to the specialist centre. Conversely, small (<5cm), subcutaneous lumps are least likely to the malignant and of the 52 patients with tumours in this category, 38 (73%) were treated at the DGH compared with 14 (27%) treated at the SC. Interestingly, even in this group who should have a good outlook both for local control and survival, there were 13 LRs in the DGH group (34%) compared with one (7%) in the SC group (*P*=0.05). There was no difference in overall survival in this good prognosis group (UICC stages la and 2b).

The fact that the SC saw any of these small, subcutaneous tumours may appear surprising but many were referred for definitive treatment after having had what is known as the 'whoops' procedure. This is when a lump is excised, usually with little forethought and without a biopsy and the surgeon is then surprised when the pathologist reports it as a sarcoma (hence the term 'whoops'). The management of these patients remains unresolved although most authors now agree that wide re-excision to obtain clear margins is necessary as residual tumour will be found in anything between 30 and 60% of cases.[17–19] Any patient who was thus referred was considered to have had their definitive treatment at the SC.

We have not in this study looked at all at the quality of care given at the different centres. The SC has defined guidelines for the management of patients with STS and all cases were discussed at a multidisciplinary team meeting. All patients will have followed this protocol which includes preoperative staging for both local and distant disease prior to excision or re-excision of the tumour. Patients with large, high grade or deep sarcomas were routinely treated with adjuvant radiotherapy according to this protocol. Chemotherapy was not routinely used for this patient population but was used for palliative management. It is likely that patients treated at a DGH will not have been treated within this protocol and whilst some will have been referred to an oncologist for further management we feel that it is unlikely there would have been much difference between this patient population and that identified by Clasby *et al.* and Jane *et al.* in their respective papers identifying very poor compliance with any accepted guidelines for care of sarcomas outside a specialist setting.[1,2]

Furthermore, we did not review the histology of the patients treated outside the specialist centre. Whilst this would have been a counsel of perfection, the ethical ramifications of potentially reclassifying patients with alleged STS into another diagnostic category is still not fully resolved. Previous experience of reviewing the pathological diagnoses of STS has shown significant errors of overdiagnosis in up to 24%.[20,21] If some patients treated for STS at a DGH did not actually have STS then this would mean that those patients would have neither had local recurrence nor disease relapse and they could only have artificially improved these rates for DGH patients.

The ability to achieve adequate margins of excision was similar in both groups even though the DGHs had a larger proportion of smaller, superficial tumours. This was, however, not then reflected in similar rates of local recurrence. A positive margin at a DGH confers a 45% risk of LR compared with a 33% risk at the SC. It is possible that this is because of the use of other adjuvants at the SC such as radiotherapy but we did not investigate this as part of the study. What was even more concerning, however, was the observation that even with allegedly clear margins at the DGH the local recurrence rate was 39% compared to 12% at the SC. This could be because of a false sense of security being produced by the pathologist's report of clear margins and consequent lack of further adjuvant treatment. Again, review of the pathology reports for accuracy, both in terms of diagnosis and assessment of margin was outside the breadth of this study.

We have shown that local control is dramatically better at the SC compared to patients treated at the DGHs and this is true for virtually all categories. Whilst we are not happy with a local relapse rate of 19% at the SC this is equivalent to those in many series. We feel however that a 40% local failure rate at DGHs is unacceptable and will have undoubtedly resulted in significant further morbidity, hospital admissions and treatment although we have made no attempt to quantify this.

The overall survival of patients in this study population was 58% at 5 years which is towards the lower limit of those published results.[1,4,16] We have shown that the same prognostic factors are significant in this population as in most other series of STS patients. Centre of treatment does not reach significance on univariate analysis but this can be explained by the disproportionate number of small, low grade and subcutaneous tumours treated at the DGHs patients who should have a better prognosis. Centre of treatment does, however, become significant on multivariate analysis when these factors are taken into account. The reason for the difference is not clear from this study. One might think that the increased local recurrence rate could be responsible for this but the role of local recurrence in overall survival remains controversial with no clearcut evidence that local relapse per se is an independent poor prognostic factor.[22,23]

We have investigated the significance of LR in this cohort of patients and have found that the presence of local relapse by itself would appear to be significant in predicting overall survival on univariate analysis ($P<0.0001$). If, however, one uses LR as a time-dependent variable (i.e., only including patients who developed local recurrence who did not already have metastases), it is no longer significant.

We conclude that this study has shown nearly identical findings to that of Gustafson in that STS treated at DGHs have a much higher rate of local relapse than those treated at the centre.[7] We have also identified for the first time that there may also be a small survival advantage to being treated at a specialist centre. We would recommend that clear network paths should be developed so that all suspicious lumps should be screened to rule out the possibility of malignancy and that any patient with a soft tissue sarcoma should be treated within a defined protocol to minimize the risk of local recurrence and maximize the chances of survival. At this point in time, this would mean referral to a specialist treatment centre for appropriate review of pathology and advice on the role of surgery, chemotherapy and radiotherapy.

### Acknowledgements

We would like to thank the Regional Cancer Intelligence Unit, and in particular Cheryl Livings who provided much support and helpful advice. Also the Caldicott Guardians of the various NHS Trusts who gave permission for data to be used for this project.

### References

1. Clasby R, Tilling K, Smith MA, Fletcher CDM. Variable management of soft tissue sarcoma: regional audit with implication for specialist care. *Br J Surg* 1997; 84: 1692–6.
2. Jane MJ, Hughes PJ. Disease incidence and results of extremity lesion treatment: Mersey Region soft tissue sarcomas (1975–1985). *Sarcoma* 1998; 2: 89–96.

3. Johnson C, Pynsent PB, Grimer RJ. Clinical features of soft tissue sarcomas. *Ann R Coll Surg Engl* 2001; 83: 203–5.
4. Bauer HCF, Trovik CS, Alvegard TA, Berlin O, Erlanson M, Gustafson P, *et al.* Monitoring referral and treatment in soft tissue sarcoma: a study based on 1,851 patients from the Scandinavian Sarcoma Group Register. *Acta Orthop Scand* 2001; 72: 150–9.
5. Rydholm A. Improving the management of soft tissue sarcoma (Editorial). *Br Med J* 1998; 317: 93–4.
6. Referral Guidelines for suspected cancer. www.doh.gov.uk/cancer/referral.htm (accessed August 2003).
7. Gustafson P, Dreinhofer E, Rydholm A. Soft tissue sarcoma should be treated at a tumor centre. *Acta Orthop Scand* 1994; 65: 47–50.
8. Rydholm A, Berg NO, Persson BM, Akerman M. Treatment of soft tissue sarcoma should be centralised. *Acta Orthop Scand* 1983; 54: 333–9.
9. Kapiteijn E, van de Velde CJ. Developments and quality assurance in rectal cancer surgery. *Eur J Cancer* 2002; 38: 919–36.
10. Sainsbury R, Haward B, Rider L, Johnston C, Round C. Influence of clinician workload and patterns of treatment on survival from breast cancer. *Lancet* 1995; 345: 1265–70.
11. Robertson AG, Robertson C, Soutar DS, Burns H, Hole D, McCarron P. Treatment of oral cancer: the need for defined protocols and specialist centres. Variations in the treatment of oral cancer. *Clin Oncol* 2001; 13: 409–15.
12. Kaplan EL, Meier P. Nonparametric observations from incomplete observations. *J Am Stat Assoc* 1958; 53: 457–81.
13. Cox DR, Regression models and life tables. *J R Stat Soc* 1972; 34: 187–220.
14. Abacus Concepts, Inc, Berkely, CA, 1996.
15. Fleming ID, Cooper JS, Henson DE *et al.* (eds). AJCC Cancer staging Manual, 5th edn. Philadelphia: Lippincott-Raven, 1997; 152–155.
16. Pisters PW, Leung DH, Woodruff J, Shi W, Brennan MF. Analysis of prognostic factors in 1,041 patients with localized soft tissue sarcomas of the extremities. *J Clin Oncol* 1996; 14: 1679–89.
17. Noria S, Davis AS, Kandel R, Levesque J, O'Sullivan B, Wunder J, Bell R. Residual disease following unplanned excision of soft tissue sarcoma of an extremity. *J Bone Joint Surg Am* 1996; 78: 650–5.
18. Giuliano AE, Eilber FR. The rationale for planned reoperation after unplanned total excision of soft tissue sarcomas. *J Clin Oncol* 1985; 3: 1344–8.
19. Goodlad JR, Fletcher CD, Smith MA. Surgical resection of primary soft tissue sarcoma. Incidence of residual tumor in 95 patients needing re-excision after local resection. *J Bone Joint Surg Br* 1997; 79: 171–2.
20. Harris M, Hartley AL, Blair V, Biorch JM, Banerjee SS, Freemont AJ, McLure J, McWilliam LJ. Sarcomas in North west England: histopathological peer review. *Br J Cancer* 1991; 64: 315–20.
21. Presant CA, Russell WO, Alexander RW, Fu YS. Soft tissue and bone sarcoma histopathology peer review: the frequency of disagreement in diagnosis and the need for second pathology opinions. *J Clin Oncol* 1986; 4: 1658–61.
22. Barr LC, Stotter AT, A'Hern RP. Incidence of local recurrence on survival: a controversy reviewed from the perspective of soft tissue sarcoma. *Br J Surg* 1991; 78: 648–50.
23. Gustafson P, Rooser B, Rydholm A. Is local recurrence of minor importance for metastases in soft tissue sarcoma? *Cancer* 1991; 67: 2083–6.
